# Performance of Mortars Made Using Ternary Binders with Addition of Slag, Fly Ash and Limestone Exposed to a Real Environmental Condition Compatible with Exposure Class XC3

**DOI:** 10.3390/ma14205937

**Published:** 2021-10-10

**Authors:** Javier Ibáñez-Gosálvez, Teresa Real-Herraiz, José Marcos Ortega

**Affiliations:** 1 Departamento de Ingeniería Civil, Universidad de Alicante, Ap. Correos 99, 03080 Alicante, Spain; javier.ibanez@ua.es; 2 Instituto de Matemática Multidisciplinar, Universidad Politécnica de Valencia, Camino de Vera s/n, 46022 Valencia, Spain; tereaher@upv.es

**Keywords:** ternary binders, real condition exposure, class XC3, ground granulated blast furnace slag, carbonation, fly ash, limestone, microstructure, mechanical properties, durability

## Abstract

The use of eco-friendly cements prepared with ternary binders could contribute to improving the sustainability of cement production. However, their use for manufacturing commercial cements is very low, at least in Spain. The purpose of this research is to study the behavior in the long term of mortars made with ternary binders which incorporated ground granulated blast furnace slag, fly ash, and limestone, exposed to environmental conditions compatible with the specifications of exposure class XC3 of the Eurocode 2, compared to mortars without additions and mortars with binary binders. The exposure station was placed in an underground floor of a building used as a garage with circulation of vehicles and moderately high CO_2_ concentration. The ternary and binary binders verified the prescriptions of cement type CEM II/B. The microstructure was characterized using mercury intrusion porosimetry and electrical resistivity. Water absorption, diffusion coefficient, carbonation depth, mechanical strengths, and ultrasonic pulse velocity were determined. A loss of microstructure refinement with time was noted for all the analyzed binders, probably related to the development of carbonation and drying shrinkage. The binary mortars with slag and fly ash and the ternary binder which combined them showed the best mechanical performance at 250 days.

## 1. Introduction

Reaching a more environmentally sustainable cement production is currently one of the main aims of the cement industry to increase its contribution to global sustainable development goals [1]. Between the different strategies for achieving this objective, the use of additions for replacing clinker has become increasingly popular [2,3,4]. The incorporation of these additions into blended cements provides environmental advantages, such as the reduction of CO_2_ emissions and the reduction of energy consumption throughout the cement manufacturing process [5], because a lower quantity of clinker is required. Additionally, the majority of these additions are wastes originating in other industrial sectors [6,7,8], so their reutilization also helps to solve the problem of their storage in landfills or specific installations, avoiding other environmental impacts. The use of additions may also have an influence in the development of the pore network and properties of cement-based materials [7,9,10,11]. For that reason, the effects of additions for developing eco-friendly cements and their applicability in real construction are currently a wide field of investigation.

In relation to the different standardized additions which are available, among the most commonly used are ground granulated blast-furnace slag and fly ash [5,12]. Many studies [7,13,14,15] have shown that these additions provide a better performance in cementitious materials in comparison with those made using ordinary Portland cement without additions. On one hand, for slag, this adequate behavior is linked to the development of hydration reactions of this addition, forming new CSH phases, which would produce a more refined pore network [7,13,16]. On the other hand, the fly ash has pozzolanic activity, which means that it is able to react with portlandite produced along the clinker hydration, resulting in the formation of additional hydrated products, which entails an increase of the pore network refinement of mortars and concretes [9,14,16,17,18]. The microstructure refinement produced by both additions improves the performance of cement-based materials, such as their resistance to chloride ingress [18,19,20,21,22,23] and sulphate attack [18,24], as well as their permeability [25]. Slag and fly ash additions belong to the group of active additions, due to their hydraulic and pozzolanic activity previously explained. Limestone is another popular addition which is widely used in cement production for substituting clinker. This addition lacks hydraulic or pozzolanic activity, so it mainly has a filler effect in cement-based materials, which also influences their microstructure and properties [26,27].

Currently, the use of additions in commercial cements is generally focused on binary binders [28], in which clinker is partially replaced by one addition. Despite that, the standards which regulate the manufacture of commercial cements [12] permit the use of ternary binders. In these binders, clinker is substituted in part by two additions. However, traditionally, the use of ternary binders in the production of commercial cements is still very low, at least in Spain [28,29]. The presence of two additions in these binders could improve the performance of cement-based materials, caused by the probable synergistic effects of blending them [30,31,32,33]. Subsequently, to analyze the performance of cement-based materials which incorporate ternary binders [26,34,35,36], focusing on their use for commercial cement manufacture, could constitute an interesting field of research in order to develop additional options to lessen the harmful pollutant effects of cement manufacture.

Within the field related to the real applications of commercial cements with additions, their behavior in construction elements could be influenced by the different environmental conditions to which they are exposed depending on their geographical location [20,37]. In this regard, the active additions seemed to be more sensitive to variations in the hardening conditions, in particular the temperature and relative humidity, because the changes in these parameters could influence the development of hydration and pozzolanic reactions [38,39,40]. With respect to the characterization of the performance of eco-friendly cementitious materials exposed to real in-situ environments [19,20,41,42,43,44], in the majority of them, binary binders were studied and the results obtained in general showed certain changeability based on the climate conditions, as well as with the presence of harmful agents in the exposure site. Therefore, to analyze the behavior of cementitious materials made using ternary binders subject to real in-situ conditions may be interesting in order to evaluate if they are adequate for being used in commercial cement production.

Therefore, the purpose of this research is to study the behavior in the long term of mortars made with ternary binders, which incorporated the additions of ground granulated blast furnace slag, fly ash, and limestone, exposed to environmental conditions compatible with the specifications of exposure class XC3, according to the Eurocode 2 [45], regarding their microstructure, durability, and mechanical properties. The mortars were exposed to the real environment of an underground floor of a building, used as a garage with moderate circulation of vehicles, with a moderately high concentration of CO_2_. This condition would be a frequent service environment of cement-based materials used in buildings. On the other hand, the additions of ground granulated blast furnace slag, fly ash, and limestone were selected for preparing the ternary binders because they are mostly used in the manufacture of blended commercial cements in Spain. In addition, the analyzed ternary binders accomplished the prescriptions for a standardized commercial cement type CEM II/B-M, according to Spanish and European standard UNE-EN 197-1 [12], in order to facilitate the possible real practical application of the obtained results by cement manufacturers. The performance of mortars prepared with ternary binders has been compared to that noted for reference mortars made using ordinary Portland cement without additions, as well as with other mortars prepared with binary binders, incorporating only one of the analyzed additions as clinker substitution.

## 2. Materials and Methods

### 2.1. Materials and Sample Preparation

The materials tested were mortars made with different binders. The first one was the reference mortar, prepared with an ordinary Portland cement without additions, type CEM I 42.5 R according to Spanish and European standard UNE-EN 197-1 [12]. This reference mortar was designated as REF in the results section.

With respect to mortars which incorporated additions, firstly, three different binary binders were studied, in which 30% of CEM I 42.5 R cement was substituted by ground granulated blast furnace slag, fly ash, and limestone. S, F, and L, respectively, were the designations of these binders in the results section.

Furthermore, mortars made with three ternary binders were prepared. One of these binders, named SL, included 15% ground granulated blast furnace slag and 15% limestone as cement CEM I 42.5 R substitution. In the second ternary binder, designated as SF, the previously mentioned cement CEM I 42.5 R was in part replaced by 15% ground granulated blast furnace slag and 15% fly ash. The third ternary binder consisted of incorporating 15% fly ash and 15% limestone as cement CEM I 42.5 R replacement, named FL. In Table 1, the designations of the analyzed binders and the percentage of their components have been compiled.

Limestone, ground granulated blast furnace slag and fly ash verified the specifications of the UNE-EN 197-1 standard [12] for their use in commercial cement manufacture as additions. The three additions analyzed in this work were facilitated by the company Cementos Portland Valderrivas (Madrid, Spain) and they are currently used for manufacturing the commercial blended cements produced by this company. The chemical compositions of blast furnace slag, fly ash, and limestone can be observed in Table 2.

The studied binders were selected for verifying the prescriptions for a standardized commercial cement type CEM II/B, according to the standard UNE-EN 197-1 [12]. The purpose of choosing this type of cement is related to the fact that today, type II cements [12] are mostly produced in Spain. Therefore, this could allow a more widespread real application of the results derived from this work.

The mortars were made with water to binder ratio 0.5. In addition, for all of them, the aggregate to binder ratio was 3:1. The fine aggregate used verified the prescriptions of the standard UNE-EN 196-1 [46].

Three kinds of specimens were made. The first kind of sample consisted of cylinders with dimensions 5 cm diameter and 6 cm height. The second kind was also cylinders, but now with 10 cm diameter and 22 cm height. Finally, prismatic samples with dimensions 4 cm × 4 cm × 16 cm were cast.

During the initial 24 h, the samples were stored in a chamber under an optimum laboratory condition (20 °C temperature and 95% relative humidity). Once finished that time, they were de-molded and kept in that optimum condition until 7 days, when they were moved to the real in-situ exposure site. It has been reported by several authors [20,40,47,48] that an adequate curing is important for satisfactorily developing the properties of cementitious and pozzolanic materials exposed to non-optimum and harmful conditions. In view of that, here, a 7-days curing period was selected. Lastly, the tests were performed at 28 and 250 days.

### 2.2. Environmental Exposure Condition

The real in-situ condition consisted of placing the samples in an underground floor of a building, used as a garage with moderate circulation of vehicles. This site would be compatible with the specifications of exposure class XC3 (corrosion induced by carbonation, moderate humidity) defined by the Eurocode 2 [45].

The exposure period started at the age of 7 hardening days, after ending the curing time of the samples, as explained in the previous subsection, and finished at 250 hardening days.

The average CO_2_ concentration measured in the exposure site along the studied time period was around 2000 ppm, reaching an absolute maximum value slightly higher than 5000 ppm. On the other hand, the temperatures registered were overall in the range between 18 °C and 23 °C, whereas the average relative humidity was in the interval from 65% to 70%.

### 2.3. Mercury Intrusion Porosimetry

This technique has been widely used for characterizing the microstructure of different types of materials [49,50,51]. A Poremaster-60 GT porosimeter commercialized by Quantachrome Instruments (Boynton Beach, FL, USA) was used. Prior to performing the test, the samples were dried in an oven at 50 °C for 48 h. Here, the total porosity and the pore size distributions obtained with this technique were analyzed. Regarding the pore size distributions, the next ranges of pore diameters were considered: <10 nm, 10–100 nm, 100 nm to 1μm, 1–10μm, 10 μm to 0.1 mm, and >0.1 mm [52,53]. Two measurements were performed on each series at both testing ages. Pieces taken from cylindrical specimens with 5 cm diameter and 6 cm height were tested.

### 2.4. Electrical Resistivity

Electrical resistivity is a useful parameter for obtaining additional data of the pore network of cementitious materials [54,55]. The resistivity of the mortars was obtained with the non-destructive Wenner four-point test, described in the Spanish standard UNE 83988-2 [56]. A Proceq analyzer was used to directly obtain the electrical resistivity of cylindrical specimens with 22 cm height and10 cm diameter. The measurements were performed at several ages up to 250 days. The surface of the cylinders was prepared according to the technical recommendation number 5 of Alconpat International [57], prior to each measurement. For each binder, three cylinders were tested and four measurements were performed per sample at each age.

### 2.5. Water Absorption

The water absorption after immersion was obtained according to the procedure explained in the ASTM Standard C642-06 [58]. Six pieces taken from cylinders with dimensions 5 cm diameter and 6 cm height were tested for each binder at 28 and 250 days.

### 2.6. Steady-State Chloride Diffusion Coefficient

The steady-state chloride diffusion coefficient was obtained from the electrical resistivity of the water-saturated samples. The electrical resistivity was measured according to the procedure explained in Section 2.4. Before the measurements, the specimens were saturated in water along 24 h following the standard ASTM C1202-97 [59]. For each series, three cylindrical specimens with 22 cm height with 10 cm diameter were tested at 28 and 250 days. Four measurements were performed per sample at both testing ages. Lastly, the steady-state diffusion coefficient was calculated with the following equation [60]:

(1) $$D_{S}=\frac{2\times 10^{-10}}{\rho }$$

where D_s_ is the chloride steady-state diffusion coefficient through the sample (m^2^/s) and ρ is the electrical resistivity of the specimen (Ω·m).

### 2.7. Carbonation Depth

The carbonation front depths in the mortars were obtained following the RILEM recommendation CPC-18 [61]. Pieces extracted from the cylinders with 5 cm diameter and 6 cm height were sprayed with a 1% phenolphthalein solution. The depth of the colorless carbonated part from the external surface of the sample was measured. For each series, six pieces taken from the abovementioned cylinders were tested at 28 and 250 days.

### 2.8. Mechanical Strengths

The compressive and flexural strengths were determined following the procedure included in the Spanish and European standard UNE-EN 1015-11 [62]. For each series, three different prismatic specimens with dimensions 4 cm × 4 cm × 16 cm were tested at 28 and 250 days.

### 2.9. Ultrasonic Pulse Velocity

The ultrasonic pulse velocity (UPV) constitutes a helpful parameter for getting additional data about the mechanical behavior of the material [63]. This parameter was obtained according to the standard UNE-EN 12504-4 [64]. In this work, the propagation time of the ultrasonic waves was determined in the largest dimension of the sample (160 mm) with direct transmission, using a Pundit Lab model commercialized by Proceq company (Schwerzenbach, Switzerland). Contact transducers which emitted ultrasonic pulses at 54 kHz were attached to the top and bottom base sides of the samples with a coupling gel. The UPV was calculated from the propagation time and the length of the sample. This parameter was obtained at several hardening times until 250 days. At each age, for the same mortar series, three prismatic specimens with dimensions 4 cm × 4 cm × 16 cm were tested and three determinations were performed per specimen.

## 3. Results

### 3.1. Mercury Intrusion Porosimetry

**Figure 1:**
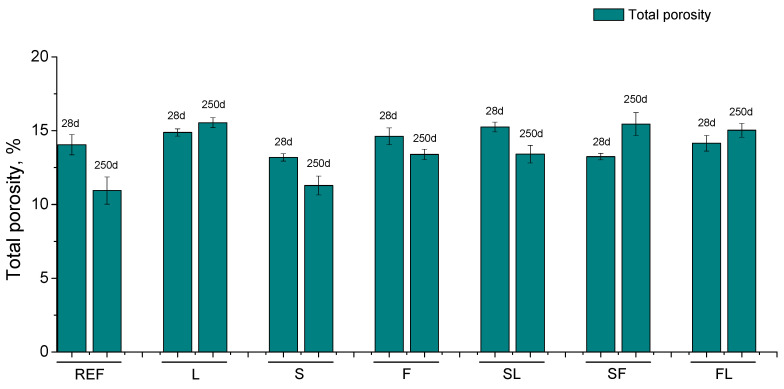
Results of total porosity for the studied mortars. In this figure and in the next figures, the mean value of the parameter obtained for each type of mortar is represented. The error bars represent the standard deviation.

Regarding the mercury intrusion porosimetry results, the total porosities noted for the binders analyzed at 28 and 250 days are shown in Figure 1. At 28 days, this parameter was relatively similar for all the mortars. Between 28 and 250 days, a reduction in total porosity was observed for REF, S, F, and SL mortars, whereas it increased for L, SF, and FL mortars. At the last age studied, the lowest total porosities were noted for REF and S binders, followed by F and SL ones. Finally, the highest values of this parameter at 250 days were noted for L, SF, and FL mortars.

**Figure 2:**
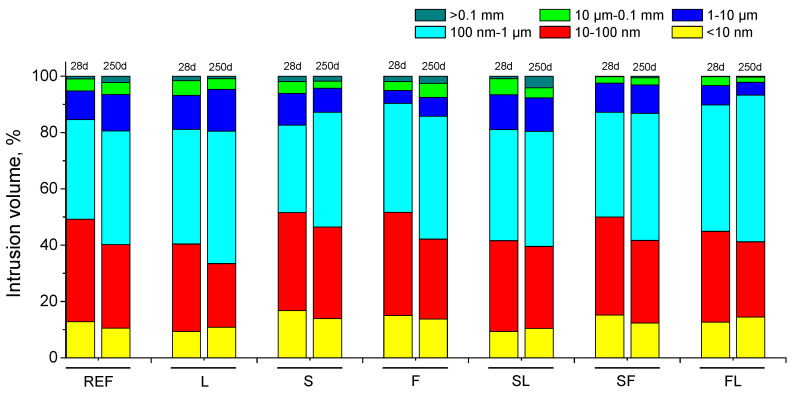
Pore size distributions noted for the analyzed binders.

The pore size distributions are depicted in Figure 2. At 28 days, the relative volume of finer pores (diameters in the ranges <10 nm and 10–100 nm) was higher for S, F, and SF binders compared to REF ones, being lower for the rest of series analyzed. In general, the proportion of pores with diameters lower than 100 nm decreased for all the mortars studied from 28 to 250 days. Despite that, the percentage of pores with sizes lower than 1 µm at 250 days was higher for S, F, SF, and FL mortars in comparison with reference specimens. This percentage was similar at that age for REF, L and SL mortars, although the REF and SL series showed higher relative volume of pores lower than 100 nm compared to L mortars.

### 3.2. Electrical Resistivity

**Figure 3:**
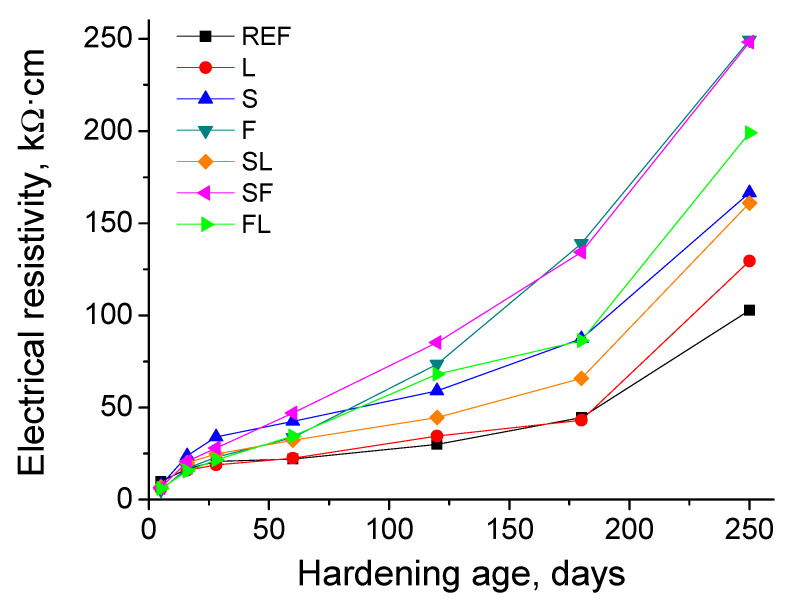
Electrical resistivity results for the different types of mortars tested.

The results of electrical resistivity are represented in Figure 3. In general, this parameter rose with age for the different types of mortars analyzed. Around 28 days, the highest values of electrical resistivity were noted for S mortars, followed by SF ones. From then, mortars with fly ash (F, FL, and SF) developed a noticeable growth of the electrical resistivity, with F and SF mortars showing the highest values of this parameter at 250 days, followed by FL binder. The other binary and ternary binders with slag (S and SL) presented lower resistivity values in the long term, compared to those specimens with fly ash. At 250 days, this parameter was very similar for both S and SL series. However, the increase with time of the electrical resistivity was slower for SL mortars in comparison with S ones. On the other hand, from 28 to 250 hardening days, REF and L mortars showed the lowest resistivity values, and the increasing rate with time of this parameter was also lower compared to the other binders studied.

### 3.3. Water Absorption

**Figure 4:**
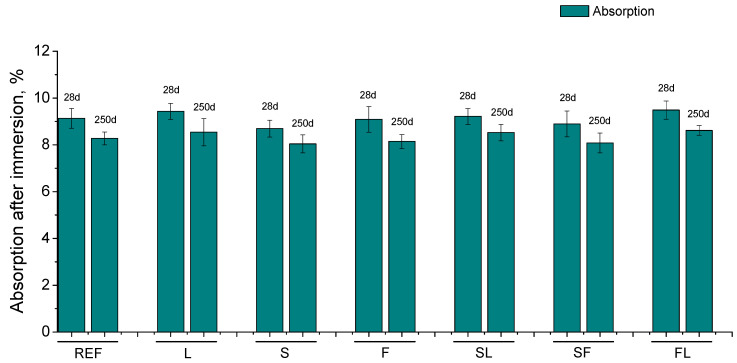
Results of absorption after immersion noted for the studied series.

The results of water absorption after immersion are depicted in Figure 4. A slight decrease with time of this parameter was noted for all the mortars studied. In general, few differences between the analyzed binders were observed at 28 and 250 days regarding the water absorption after immersion.

### 3.4. Steady-State Chloride Diffusion Coefficient

**Figure 5:**
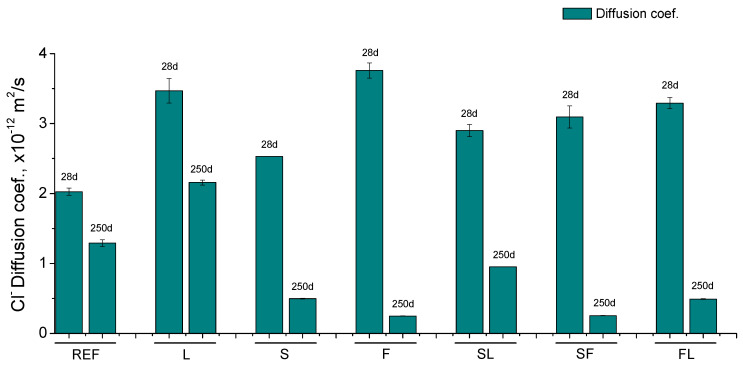
Steady-state chloride diffusion coefficient results obtained for the analyzed mortars.

The results of steady-state chloride diffusion coefficient obtained from sample’s resistivity for the analyzed mortars can be observed in Figure 5. All the binary and ternary binders studied showed higher values of this parameter at 28 days compared to the reference specimens. The highest diffusion coefficients at that age were noted for F and L series, followed by the three ternary binders analyzed (SL, SF, and FL series). On the other hand, the lowest 28-days value of this parameter for mortars with additions corresponded to the S series. Between 28 and 250 days, the diffusion coefficient decreased for all the mortars studied, and this reduction was more noticeable for those which incorporate at least one active addition in the binder (S, F, SF, SL, and FL series). The lowest coefficient at 250 days was noted for F and SF mortars, closely followed by S and FL ones, while it was slightly higher for SL series. In addition, for all the binary and ternary binders already mentioned, the diffusion coefficient was lower at 250 days in comparison with reference mortars. Lastly, at that last age, L mortars presented the highest value of this coefficient.

### 3.5. Carbonation Front Depth

**Figure 6:**
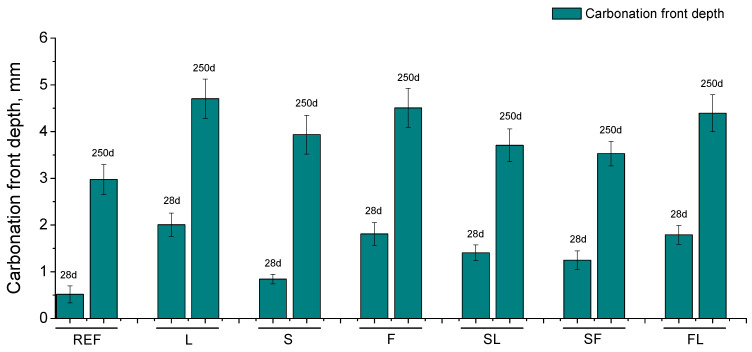
Carbonation front depths observed at 28 and 250 days for the characterized binders.

The depths of carbonation front obtained for the studied binders are represented in Figure 6. Reference mortars showed the lowest carbonation front depths at 28 days, followed by S ones, whereas these depths were higher for the other binders studied, but with slight differences between them. From 28 to 250 days, the carbonation depths increased for all the analyzed mortars. At 250 days, this parameter was again lower for REF series, compared to the binders with additions. The highest carbonation depths at that age were noted for the binary binders, particularly for L mortars. Regarding the ternary binders (SL, SF, and FL series), they presented slight lower depths than binary ones.

### 3.6. Mechanical Strengths

**Figure 7:**
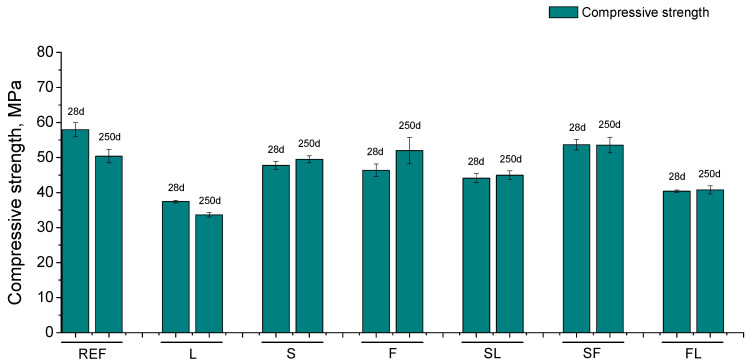
Compressive strength results noted for the analyzed mortars.

The compressive strength results are depicted in Figure 7. This parameter was lower for the mortars with additions at 28 days in comparison with REF specimens. With respect to the binders with active additions, the highest compressive strength in the short term was observed for SF mortars, whereas this parameter was barely lower for S, F, and SL series, followed by FL ones. At 28 days, the smallest compressive strength corresponded to L mortars. Between 28 and 250 days, the compressive strength decreased for the REF and L series, while for binders with at least one active addition, it remainedpractically constant (S, SL, SF, and FL series) or even increased (F series). The S, F, and SF mortars showed very similar compressive strength at 250 days compared to REF mortars. Nevertheless, at the abovementioned age, the ternary binders with limestone (SL and FL series) had lower values than reference mortars, although their compressive strength was higher than that observed for the binary binder with only limestone as addition (L series).

**Figure 8:**
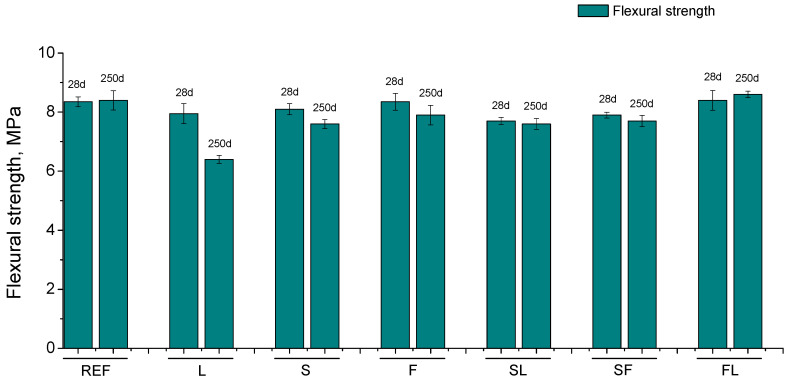
Flexural strength results for the different mortars tested.

In relation to the flexural strength, its results can be observed in Figure 8. Scarce differences have been noted in this parameter between the mortars tested, being in the range from 7.5 to 8.5 MPa for most of them at the studied hardening ages. This strength hardly changed with time for REF, SL, SF, and FL series, and it decreased slightly for S and F ones. The most noticeable fall of flexural strength from 28 to 250 days was observed for L mortars, showing the lowest value of this parameter at 250 days of all the studied series. At that last testing age, the highest flexural strength was noted for REF and FL mortars, closely followed by the S, F, SL, and SF series.

### 3.7. Ultrasonic Pulse Velocity

**Figure 9:**
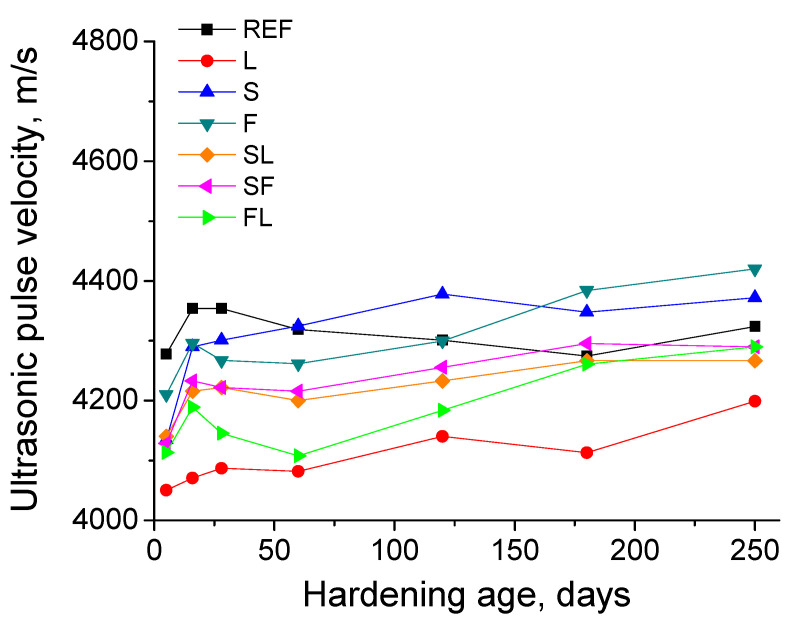
Ultrasonic pulse velocity results for the analyzed binders.

The evolution of the ultrasonic pulse velocity (UPV) is represented in Figure 9. In general, the main increase of this parameter for most of the analyzed binders was observed in the very short term. At initial hardening times, REF mortars showed higher UPV than the other series studied. With respect to the mortars with active additions, at those early ages, the binary binders S and F presented slight greater values of this parameter than the ternary binders SF, SL, and FL. At later ages, the UPV for the mortars which incorporate active additions overall rose with time, although with a relatively slow rate. In contrast, this parameter slightly decreased in the long term for reference mortars. At 250 days, the UPV was higher for S and F series compared to REF ones. Moreover, for the tested ternary binders (SF, SL, and FL series), it was similar to reference mortars. During the studied hardening time, the lowest values of UPV parameter were noted for L mortars.

## 4. Discussion

### 4.1. Microstructure Characterization

Firstly, in relation to total porosity results (see Figure 1), the scarce differences at 28 days in this parameter between the different mortars analyzed could be related to the environmental relative humidity (RH) in the exposure location of the samples. As was explained in Section 2.2, the average RH registered in the exposure station was in the range 65–70%. This RH was relatively high, although it was lower than the RH of an optimum standardized laboratory condition (95–100%). Therefore, this lower RH in the environment could affect the slag and clinker hydration reactions [39,65], because they need the presence of water to be developed [66,67]. Then, with a lower availability of water in the environment, these hydration reactions would slow down [19,39,65], giving as a result that the total porosity differences were less noticeable in the short term comparing the binders analyzed. Furthermore, this could affect the binders with fly ash too, because the development of pozzolanic reactions of fly ash could also be slowed down by a lower environmental RH [68,69], in a similar way to that explained for slag and clinker hydration [19,39,65]. In addition, the slower development of those hydration reactions could produce a delay in the starting of fly ash pozzolanic reactions [69], because they need the presence of enough portlandite to be developed [9,14].

The differences in pore size distributions at 28 days between the binders analyzed (see Figure 2) were not high, which could be related to the abovementioned effects of the lower environmental RH, although several effects of the studied additions in the microstructure of mortars could be observed. On one hand, the pore network was more refined at 28 days for S, F, and SF binders, as suggests their higher percentage of pores with sizes lower than 100 nm, and particularly those pores in the range <10 nm, compared to reference specimens. This could be related to the short-term influence in the microstructure of slag hydration [39,65,70] and fly ash pozzolanic reactions [9], which produced additional solid phases [7], and their effects were noticeable despite the lower RH provided by the environment. Moreover, when both slag and fly ash additions were combined in the same binder, their combined effects also gave an improvement of the microstructure refinement, at least in the short term and under the environmental conditions analyzed, as would indicate the pore size distribution of SF series at 28 days, compared to the reference one. On the other hand, the slightly less refined pore structure noted for ternary binders with limestone (SL and FL series) at short times, in comparison with the other binders with slag and fly ash, would be related to the inert character of the limestone, because it is not an active addition, without hydraulic or pozzolanic activity [71]. Therefore, the limestone addition only has a filler effect [71] and it does not produce additional solid phases, quite the opposite of what happened with slag and fly ash [7], so its influence in the microstructure is more limited. This would also explain the less refined microstructure of all the mortars studied for binary binder with limestone (L series).

Regarding the evolution with time of the pore size distributions, a loss of the microstructure refinement was observed for all the mortars studied from 28 to 250 days, as suggested by the reduction of the relative volume of finer pores size intervals (see Figure 2). On one hand, this could be due to development of the carbonation phenomenon in the mortars, produced by the CO_2_ present in the environment (see Section 2.2), as revealed by the carbonation front depth measurements (see Figure 6). Several authors [66,67,72] have reported that this coarser pore network due to the carbonation development could be related to the additional formation of silica during the decomposition of C-S-H gel caused by the exposure to CO_2_. On the other hand, the lower RH in the environment could also have an influence in the reduction of microstructure refinement with the hardening time, producing the formation of shrinkage microcracks by drying [65,67,73]. According to several works [67,74], the RH of the exposure medium greatly affects the magnitude of shrinkage. Therefore, the development of shrinkage microcracking could contribute to produce a coarser pore structure in the long term for the analyzed binders. Additionally, as has been already explained, the lower RH would make the development of hydration and pozzolanic reactions slower and more difficult once the setting water was consumed [19,39,65,68,69], so the additional solid phases formation as products of those reactions could not totally counteract the effects of carbonation and shrinkage in the microstructure. Despite that, the overall higher pore refinement observed at 250 days for ternary and binary binders with at least one active addition (slag and/or fly ash) incorporated, compared to reference specimens, would reveal their beneficial long-term effects in the microstructure [7], even under the exposure to a non-optimum real condition.

With respect to the evolution of total porosity between 28 and 250 days (see Figure 1), it would also be influenced by both shrinkage and carbonation phenomena. For several binders studied (REF, S, F, and SL series), this parameter decreased with time and this reduction was more noticeable for REF mortars. In this regard, several authors [72,75,76] have reported similar results in cement-based materials exposed to carbonation. This could be related to the formation of CaCO_3_ inside the pores, as products of carbonation development, because the CaCO_3_ occupies a higher volume than the initial hydrated phases from which it is formed, giving as a result a reduction of porosity [66]. The higher reduction of total porosity noted for REF mortars would also be in keeping with other researchers [72]. These reference mortars had greater content of clinker than those with additions. Therefore, the reference mortars would have a higher availability of portlandite (formed by clinker hydration) for reacting with the environmental CO_2_, so a greater amount of CaCO_3_ as a consequence of the carbonation process would be produced [72], giving as a result the more notable reduction of total porosity noted for REF series. Furthermore, the loss of microstructure refinement due to carbonation, already discussed in terms of the pore size distribution results, while a reduction of total porosity was simultaneously produced, would agree with other works [72,76].

Nevertheless, the possible development of shrinkage microcracking in the long term due to the lower RH in the environment would produce an increase of total porosity [65,67,73,74], as has been previously explained for the results of pore size distributions. Then, the effect of the shrinkage would counteract the decrease of porosity produced by carbonation. The more noticeable influence of shrinkage could explain the increase of total porosity with age noted for some of the analyzed binders (L, SF, and FL series).

Electrical resistivity is a useful parameter for getting information related to the pore connectivity and the changes in the pore structure. For all the studied mortars, an increase of the electrical resistivity with hardening time was observed (see Figure 3), which would suggest a progressive microstructure refinement [77], with a rise of the relative volume of finer pores. This result would not be in agreement with those obtained with mercury intrusion porosimetry, particularly with the pore size distributions previously discussed, which revealed an overall coarsening of the microstructure with age. Firstly, the saturation degree of the material is one of the most important factors which can affect the electrical resistivity values [57,77]. This saturation degree changes when a drying of the material is produced, for example under non-optimum RH environments. Several works have concluded that the electrical resistivity considerably increases as the saturation degree of the material decreases [78,79]. In the analyzed environment, the average RH was around 65% (see Section 2.2), so it would produce a progressive drying of the samples, which could explain the gradual rise with time of electrical resistivity noted for the mortars.

Another possible factor that could have contributed to the differences between electrical resistivity and porosimetry results would be related to the characteristics of each technique and the geometry of the samples used for each one. On one hand, small pieces taken from cylindrical specimens with 5 cm diameter and 6 cm height were tested with mercury intrusion porosimetry. On the other hand, the electrical resistivity was measured with the Wenner four-point test on cylinders with 22 cm height and 10 cm diameter. In the case of cylindrical specimens with 5 cm diameter and 6 cm height, due to their relatively small volume, after 250 days of exposure, the development of their microstructure would be more globally affected by the environmental conditions, and as a consequence by the abovementioned harmful processes developed in this environment, previously described. For the cylinders with 22 cm height and 10 cm diameter, their higher size and volume would entail that the external part of the sample would be more influenced by the environment, whereas its influence would be lower and delayed in the core of the sample. Then, the development of the microstructure towards finer pores could be higher in the core part of the sample, because the effects of environmental drying would affect this part with a lesser degree, allowing a greater development of hydration and pozzolanic reactions [39,65,66,67]. The electrical resistivity measurements provide more global information about the microstructure of the sample [77] compared to mercury intrusion porosimetry, so these possible differences in the pore network between different parts of the specimen could explain the lack of coincidences between the resistivity measurements and pore size distributions.

Despite the abovementioned arguments, all the samples used for measuring the electrical resistivity were subject to the same environment during the studied time period, so the results of this parameter for the analyzed mortars can be compared. At early ages, the greater resistivity values showed by S and SF binders, could be related to the hydration of slag [7], already explained. The low resistivity noted in the short term for binary and ternary binders with fly ash (F and FL series) and their progressive growth with time (F, SF, and FL series), even overtaking the values of the other binders with slag (S and SL series) at the latest testing age, would show the effects of fly ash pozzolanic reactions [9,80], reducing the pore size, as well as the delay of their starting compared to slag and clinker hydration, previously mentioned in the discussion of total porosity results at 28 days. Finally, the greater electrical resistivity noted for binders which incorporate at least one active addition (fly ash and/or slag) would be in keeping with other authors [77]. This would indicate a higher proportion of pores with small sizes in their porous network, agreeing with results of porosimetry, which generally showed a higher pore refinement in those mortars in contrast with REF mortars, and particularly with L ones.

### 4.2. Durability and Mechanical Parameters

Regarding the durability-related parameters, the highest values of the steady-state chloride diffusion coefficient noted at 28 days (see Figure 5) for the binary binder with fly ash (F series) would be related to the abovementioned delay of the initiation of fly ash pozzolanic reactions [9,14], compared to slag and clinker hydration, in which the lower RH in the environment could also have an effect. This delay was also noticeable in the ternary binders with fly ash (FL and SF series), although their diffusion coefficient at 28 days was lower than that noted for F series, probably due to the influence of the other addition present in these binders, such as the filler effect of limestone [26,71] and the slag hydration [7,66]. The lowest diffusion coefficients noted for REF and S mortars at 28 days could be explained in terms of clinker and slag hydration, particularly their sooner starting [39,65], despite the lower environmental RH. In the case of binary binder with limestone (L series), the high diffusion coefficient in the short term could be related to the lack of hydraulic or pozzolanic activity of this addition [26,71], already discussed for pore size distributions results. The non-active character of this addition would also explain the higher coefficient at 28 days noted for SL binder in comparison with S one.

With respect to the evolution of the diffusion coefficient, a decrease of this parameter from 28 and 250 days was observed. This tendency would be overall in agreement with the rise with time of electrical resistivity, although it would not coincide with the evolution of pore size distributions. As was described in Section 2.6, the steady-state chloride diffusion coefficient was determined from the electrical resistivity of water-saturated samples. These samples were cylinders with 22 cm height and 10 cm diameter, similar to those used for following the changes in the electrical resistivity in non-saturated samples. Therefore, part of the arguments previously given to justify the differences between the porosimetry and resistivity results would also be valid for explaining the evolution of diffusion coefficient. On one hand, the effect of saturation degree of the material would not be considered in the results of this parameter because water-saturated specimens were used for measuring the electrical resistivity, from which the diffusion coefficient was calculated. On the other hand, the different development of the microstructure in the external and core part of the sample, more notable in those specimens used for studying the electrical resistivity and the diffusion coefficient than in those used for taking the pieces tested with porosimetry, could be compatible with the diffusion coefficient results obtained. Then, the lower influence of the environment, particularly the drying process produced by the lower RH, would allow the pore network to become more refined in the inside part of the samples, giving as a result a lower global chloride diffusion coefficient, despite being superficial parts of the sample with a coarser microstructure and more affected by the exposure condition and its harmful processes.

It is interesting to highlight that all the binary and ternary binders with slag and/or fly ash tested showed lower diffusion coefficients at 250 days than reference mortars under the studied environment. The appreciable fall of this parameter for samples with fly ash (F, SF, and FL series) would show the effect of the pozzolanic activity of fly ash [9,14] in the pore size reduction in the long term, previously explained. The slag hydration produced a similar effect [7], also entailing an important reduction of the diffusion coefficient with time, as suggested by the results of the S, SF, and SL series. In REF and L mortars, the clinker hydration would contribute to reducing this parameter, although their less refined microstructure would entail the higher diffusion coefficients noted for them in the long term.

In relation to the water absorption after immersion (see Figure 4), scarce differences between the studied binders have been noted at the testing ages, and a slight decrease of this parameter was observed for all of them from 28 to 250 days. This result would not coincide with those previously discussed, particularly the similar values for all the binders. This lack of differences could be related to the experimental procedure described in the ASTM Standard C642-06 [58], which was followed to determine the water absorption. The first step of this procedure consisted of drying the samples in an oven at a temperature of 100 to 110 °C for not less than 24 h. This conditioning of the samples could induce the formation of shrinkage cracks in the mortars [25,67,73], which would homogenize the behavior of the mortars regarding the water absorption by immersion (the samples were water-saturated once finished the drying process in a second step of the experimental procedure [58]). This would mask and notably reduce the effects produced by the exposure environment in each binder, giving the uniform result obtained in this parameter.

The higher carbonation depths observed at 28 and 250 days for the binary and ternary binders studied in comparison with reference specimens (see Figure 6) would agree with other authors [66,67,72,76]. This could be explained in relation to the lower content of portlandite in those mortars prepared with binary and ternary binders [67], which would imply a faster carbonation of the material for the same concentration CO_2_ in the environment, reaching higher depths in the samples, as has been noted. Comparing the mortars with additions, at both ages studied, the carbonation depth was slight higher for the L, F, and FL series. In the case of fly ash, this could be related to its pozzolanic activity, which would entail a lower presence of portlandite, because it is consumed in the pozzolanic reactions [9,14,66], giving as result the deeper carbonation observed, being in keeping with the results pointed out by other authors [76]. Regarding the limestone, it has no pozzolanic activity, so the greater carbonation depths, particularly for the L binder, could also be explained in terms of less pore refinement produced by this addition [67]. The increase of carbonation front depths between 28 and 250 days for all the binders would be expected due to the exposure to the analyzed environment (see Section 2.2), in an underground garage with moderate circulation of vehicles. Finally, as has been explained in the discussion of mercury intrusion porosimetry results, the progressive development of carbonation produced a loss of microstructure refinement with time, probably due to the reaction of C-S-H compounds with the environmental CO_2_, producing silica formation, as has been reported by several authors [66,67,72].

The effects of the exposure environment were also noticeable in the compressive strength (see Figure 7). In general, lower values of this parameter were obtained for mortars which incorporated limestone (L, SL, and FL series). As was explained in the microstructure characterization, this would be due to the fact that this addition does not have hydraulic or pozzolanic activity [71], having a negative effect on the compressive strength of the materials, even for the ternary binders in which it was combined with slag or fly ash. It is also interesting to highlight the good performance of SF binders in relation to this parameter, which would be due the synergetic effects combining two active additions in the binder [15]. The flexural strength showed a similar order of magnitude for all the mortars (see Figure 8), which would suggest a similar influence of the environment, independently of the binder, with only the lower values of this parameter noted for L mortars being remarkable, attributable to the abovementioned non-active character of this addition.

The scarce change or the decrease of both compressive and flexural strengths with exposure time would be overall in agreement with the pore size distributions, which showed a progressive reduction of pore refinement. This would be related to the harmful processes developed in the environment. On one hand, the lower RH in the environment would make the development of hydration and pozzolanic reactions more difficult [19,39,65,68,69], limiting the strength improvement with time. This lower RH would also produce the formation of drying shrinkage, which could lead to the formation of microcracks, which would contribute to the scarce rise or worsening of the mechanical performance of the materials [40,73,81]. On the other hand, the carbonation process, already described, would also have an influence in the recorded strength results, because their development also leads to a loss of the mechanical behavior of cement-based materials [82,83]. Lastly, it is interesting to highlight the adequate compressive strength of S, F, and SF at 250 days, with very similar values to reference ones.

The ultrasonic pulse velocity (UPV) results showed similarities with the mechanical strength ones. In the short term, the UPV was higher for reference mortars (see Figure 9), whereas it decreased with time after 28 days, in keeping with the results of compressive strength. This would indicate the initial effects of clinker hydration, closing the microstructure and improving the mechanical behavior of the material, and thus raising the UPV, as well as the damaging influence of the environment in the long term, which was revealed by the reduction with time of this parameter. The rest of the binders studied showed a similar trend, with a UPV increase in the very short term, linked to the early development of hydration and pozzolanic reactions, which reduced the voids in the mortars [7], and keeping practically constant or barely increasing this parameter from 28 days on, due to the harmful effects of the environment, as already discussed. In the particular case of the S and F series, the UPV rose with time, but with a slower rate for F, coinciding with their compressive strength results and showing the different behaviors of slag and fly ash additions. Ternary mortars with limestone overall showed slight lower UPV values in the long term compared to reference ones, which would also agree with their lower compressive strength results. The lowest UPV was noted for L mortars, coinciding with their lowest compressive strength of all the studied binders, due to the explained influence of limestone addition [71]. Finally, S and F mortars presented greater UPV values than REF specimens at 250 days, and they were very similar for SF series compared to REF ones, so this would also be in accordance with their compressive strength results at that age.

## 5. Conclusions

The main conclusions that can be drawn from the results previously discussed can be summarized as follows:

- In the short term, the differences regarding the total porosity and the pore size distributions were not high between the studied mortars. Despite that, the pore network at 28 days was more refined for binary binders with slag and fly ash (S and F series) and for ternary binder which incorporate both additions (SF series). The presence of limestone in the binder overall reduced this pore refinement.
- A loss of microstructure refinement with time was noted for all the analyzed binders. This could be related to the development of carbonation process, due to the CO_2_ present in the environment, as well as by the possible formation of drying shrinkage microcracks in the long term caused by the lower environmental relative humidity.
- The slight differences between the studied binders regarding the water absorption after immersion would be influenced by the experimental procedure used for its determination, which would homogenize the effects of exposure environment in each binder.
- The carbonation front depths were higher for the analyzed binary and ternary binders in comparison with reference mortars. This was particularly noticeable for mortars with fly ash and limestone.
- The mechanical strengths hardly changed or even decreased with time depending on the binder, which would also be related to the harmful processes developed in the environment, such as carbonation and drying shrinkage. In addition, the ultrasonic pulse velocity results were compatible with the mechanical strength ones.
- The main novelty of this research was its analysis of the behavior of several mortars prepared with binary and ternary blended cements, which accomplished the prescriptions required for a standardized commercial cement type CEM II/B when they were exposed to environmental conditions compatible with the specifications of exposure class XC3, defined by Eurocode 2. In view of the results obtained, it is interesting to highlight that the binary and ternary binders with at least one active addition overall showed a higher pore refinement and lower steady-state chloride diffusion coefficient in the long term compared to reference mortars. In relation to the mechanical properties at later exposure times, the binary mortars with slag and fly ash (S and F series) and the ternary binder with both additions (SF series) showed the best mechanical performance, similar to reference mortars.

## Figures and Tables

**Table 1:** Designation of the mortars prepared and percentage (in weight) of the binder components.

Designation	CEM I 42.5 R	Blast Furnace Slag	Limestone	Fly Ash
REF	100%	-	-	-
F	70%	-	-	30%
S	70%	30%	-	-
L	70%	-	30%	-
FL	70%	-	15%	15%
SF	70%	15%	-	15%
SL	70%	15%	15%	-

**Table 2:** Chemical composition of the additions used in this work.

Components	Fly Ash	Blast Furnace Slag	Limestone
SiO_2_	54.40%	31.50%	2.85%
MgO	1.40%	6.98%	0.47%
K_2_O	3.12%	0.52%	0.18%
Al_2_O_3_	27.70%	10.10%	1.22%
SO_3_	0.53%	1.94%	0.10%
TiO_2_	1.05%	0.94%	0.11%
Fe_2_O_3_	8.06%	0.37%	0.54%
CaO	2.55%	46.80%	94.40%
P_2_O_5_	0.46%	0.02%	0.02%
ZnO	0.11%	-	-
MnO	0.06%	0.17%	-
Na_2_O	-	0.30%	-

## Data Availability

The data that support the findings of this study are available from the corresponding author, J.M.O., upon reasonable request.
